# Women of Color in the Health Professions: A Scoping Review of the Literature

**DOI:** 10.3390/pharmacy12010029

**Published:** 2024-02-07

**Authors:** Olihe Okoro, Omolayo Umaru, Meghana Ray

**Affiliations:** 1 Department of Pharmacy Practice and Pharmaceutical Sciences, University of Minnesota, Duluth, MN 55812, USA; ookoro@d.umn.edu; 2 Department of Pharmaceutical Care & Health Systems, University of Minnesota, Minneapolis, MN 55455, USA; umaru002@umn.edu; 3 HEED Lab, LLC, Health Analytics Network, LLC, Rockville, MD 20852, USA

**Keywords:** intersectionality, healthcare professions, women of color, pharmacy, racial/ethnic inequity, gender inequity, discrimination, professional advancement, underrepresentation

## Abstract

Women of color (WoC) in the health professions encounter challenges in advancement to higher positions, disparities in wages, discrimination, lack of expectation to achieve leadership positions, and absence of extensive support networks. Articles in the literature have addressed race and/or gender in the context of professional development. However, applying an intersectional lens or framework to better understand the contextual issues of professional development for WoC remains to be addressed. Thus, this scoping review aimed to (i) identify health professions literature that addresses disparities affecting WoC, and (ii) describe strategies and approaches to support WoC in the health professions. Methods: The literature searches were conducted in multiple databases, including PubMed and MEDLINE (Ovid); and Google and Google Scholar were used to “hand search” further articles including gray literature. Three independent reviewers reviewed and screened articles for inclusion in accordance with a guide. Search included articles on pharmacy or healthcare professions, published in English, and which met three content criteria: racial disparities/inequities, professional development/career advancement, and women or gender disparities Results: A total of 31 articles were included—medicine (17), nursing (1), pharmacy (7), other (4), and multiple health professions (2). Key findings included underrepresentation of women and minority groups, inequities in professional advancement and leadership positions for WoC, and greater dissatisfaction and attrition among minority and women professionals. Conclusion: WoC face unique and distinct challenges and barriers in their professional careers resulting from the intersectionality of not only race and gender, but also lived experiences and opportunities. Strategies to improve diversity and representation should include an intersectional framework or lens and be critically evaluated.

## 1. Introduction

Public discourse in the past few years to dismantle systemic and institutionalized racism has led to a number of resolutions and actions that aim to increase minority representation, particularly in health and science. It has been long understood that the health professions should actively recruit, retain, and promote professionals from diverse ethnic and minority groups in the context of complexity of patient care, rising costs, and technological advancements [1]. Fostering a diverse workforce improves communication, healthcare access, patient satisfaction, and problem solving for complex challenges, cultivates innovation, and decreases health disparities [2]. In the context of addressing disparities in the health workforce, there are two distinct groups of underrepresented workers—women and minorities.

The World Health Organization’s landmark report “Delivered by women, led by men: a gender and equity analysis of the global health and social workforce” calls for urgent action to address gender inequities in the health and social care workforce in order to reach universal health coverage and other sustainable development goal targets [3]. This report identified four areas that need to be addressed: gender parity in leadership; occupational segregation; decent work free from bias, discrimination, and harassment, including sexual harassment; and the gender pay gap [3].

The concentration of women who are Black, Indigenous, and people of color in low-wage health care occupations is well established [4]. Women of color (WoC) are concentrated in the most physically demanding direct care jobs (nursing aide, licensed practical nurse, or home health aide), along with support jobs like cleaning and food preparation in hospitals, nursing homes, and schools [5]. By contrast, white women are disproportionately represented in jobs with supervisory capacity, in public-relations-related jobs, or jobs with authority such as registered nurse and social worker [6,7,8]. The stratification of the healthcare workforce has historical roots in slavery, creating an exclusionary labor market that relegated WoC, and Black women in particular, to domestic work, farm work, and marginal factory jobs [9]. In the twentieth century, as the service economy emerged and expanded, care work found its way into institutionalized settings where yet again, an overwhelming proportion of the burden of low-wage jobs requiring physical labor fell on WoC.

*“Any economist will tell you that diversification is the key to a secure portfolio. Any geneticist will tell you that diversification is key to maintaining hardy species of plants and animals. But somehow, when it comes to racial politics, the virtues of diversity are lost. Diversity in health care is not about fair representation—it is about saving lives.”*—Commissioner George Strait, Associate Vice Chancellor for Public Affairs, University of California, Berkeley

Among the advanced healthcare systems, the U.S. healthcare system, though exceptional in its achievements, has barely kept up with the changing demographics, which, in turn, has led to disparities in access to treatments and treatment outcomes. The landmark 2003 Institute of Medicine report “Unequal treatment” highlighted the lower quality of health care, higher rates of illness, disability, and premature deaths among minority populations [10]. This report provided compelling and alarming data along with factors contributing to the increasing disparities in health care outcomes through cultural differences, high rates of poverty, lack of access to health care, and unemployment. Further, the report recognized the dearth of minority health professionals and recommended increasing the number of minority health professionals as a key strategy in eliminating disparities. Following on this, the 2004 Sullivan Committee report “Missing Persons: Minorities in the Health Professions” emphasized the need for leadership, commitment, and accountability at the highest levels in institutions of learning [11].

Women constitute slightly more than 50% of the U.S. population, represent approximately half of the labor force, serve as breadwinners in over 40% of homes, and control 70–80% of consumer purchasing and spending [12,13]. According to the National Academy of Sciences, “It is not talent, but unintentional biases and outmoded institutional structures that are hindering the access and advancement of women” [14]. Often, women in the health workforce are beset by the better known “*glass ceiling*” in their leadership aspirations [15]. The healthcare system in the US is not untouched by the historical injustices, prejudices, and systemic undermining of people of color.

In 1989, Kimberle Crenshaw introduced the concept of “intersectionality” in her seminal work to refer to the “compoundedness” of subordination due to multiple factors including, race, age, gender, and sexuality, among others. As Crenshaw demonstrates in her work, the experience of marginalization and discrimination by Black women is distinctly different from those experienced by white women and Black men [16]. In extrapolating this, as the literature has indicated, the experience of discrimination and marginalization is challenging and different for WoC. The stereotyping, generalizations, and presumptions of one’s race and/or ethnicity coupled with being a woman create distinct challenges for many talented individuals to overcome in order to succeed and rise in their chosen professions. This is seen in the healthcare professions in the U.S., which are significantly under-represented by professionals of color. In 2019, the healthcare diversity tracker project of the George Washington University found that Black, Latino, and Native American people were severely under-represented in the healthcare workforce [17]. In fact, in 2019, only 12.1% of the U.S. healthcare workforce was Black, 18.2% were Latinos, and native Americans accounted for a mere 0.6% [17].

These discussions have taken on a global context given that ethnic and gender inequities are not limited to the US.

Despite the global nature of this issue, there remain gaps in the scientific literature on how to address inequities and evaluate progress. We, therefore, conducted a scoping review with the aim to identify articles in the healthcare literature that address barriers and challenges WoC face in the healthcare professions along with programs or strategies aimed at addressing professional development and leadership among WoC globally. Since healthcare professions are global, we did not restrict our search criteria to the US, but also broadened the scope to include research in the literature from other countries. We further aimed to identify which of these articles use an intersectional lens or framework in their analyses. Lastly, we chose pharmacy as a case study to discuss the professional barriers and opportunities for WoC as explained in the Section 4.

## 2. Materials and Methods

In this study, we conducted a scoping review, guided by the 5-stage process outlined by Mak and Thomas [18] as follows:

Stage 1: **Identify the research question.**

The research questions for this study were: *What is the status of the existing literature on gender and race intersectionality in the healthcare professions? What are the aspects, systems, and processes of inequity that affect professional development and career advancement for WoC in the pharmacy profession (and other healthcare professions)? What strategies, tools, approaches are developed and/or recommended to address intersectionality in the workplace?*

Stage 2: **Identify the relevant literature.**

A comprehensive search was conducted using two electronic databases: PubMed and MEDLINE (Ovid). Search terms included combinations of the following keywords: *women/female, WoC/BIPOC women, professional development, career advancement, equality/equity, medicine, pharmacy, nursing, intersectionality, approach/strategy/tool, systemic racism/anti-racism/racism, and coauthor/partnered/collaborative/engaged health research.* Search was not restricted by year; and was conducted between June and August, 2023. To supplement this search, we used Google and Google Scholar search engines to identify any additional articles and gray literature (e.g., theses/dissertations) that may not have been indexed in PubMed or Medline. Inclusion and exclusion criteria were clearly defined prior to beginning the search. (see Stage 3 below).

Stage 3: **Study selection.**

**Figure 1:**
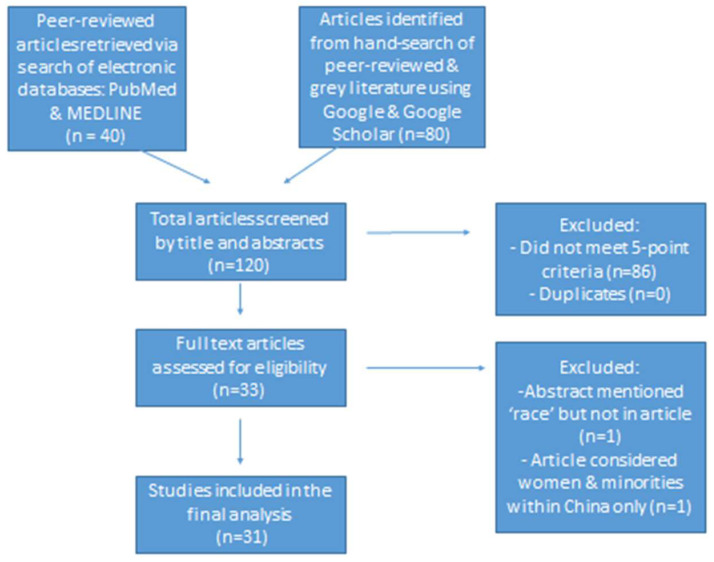
Flow-chart of the article- election process.

All retrieved articles were entered into an Excel spreadsheet and checked for duplicates. Article selection involved two steps: Initially, using title and abstract, each reviewer independently screened articles, using separate grids to ensure “blinding”. Articles about *pharmacy* or other *healthcare professions* were included in the study. The included articles had to meet 3 additional content criteria: *racial disparities/inequities, professional development/career advancement; and women or gender disparities.* If articles met all three criteria, they were included in the study. Articles that did not meet these criteria were excluded, including articles that were not published in English. Ethical approval was not required because the review is not considered human subject research. The next step involved a comprehensive review of the full article, with pairs of reviewers reviewing each paper as primary and secondary reviewers. This was followed by an assessment of consensus among reviewers regarding article recommendations (to include/to exclude). Points of non-concordance were successfully addressed by referring to stated criteria. (See Figure 1)

Stage 4: **Charting the data.**

Selected articles were extensively reviewed by 2 independent reviewers who collected and tabulated the characteristics of each selected article, and independently extracted data into predefined categories (as suggested by the guide), as well as categories that emerged. Categories for data extraction included *author names, year of publication, geographical location, study population, area of focus, study limitations, recommendations, and key relevant findings* where applicable. Relevant data, including author names, publication year, geographical location, study population, focus area, limitations, recommendations, and key findings, were extracted and tabulated as suggested by the guide. Both reviewers then met to present emerging categories, and further inclusion of article type and study design. Any discrepancy in categorization between reviewers was discussed until consensus was reached and the form was calibrated accordingly. Following this consensus, the extraction process was completed for all articles included in the study.

Stage 5: **Collating, summarizing, and reporting the results.**

We examined the aspects, systems, and processes of inequity that affect professional development and career advancement for WoC in the pharmacy profession (and other healthcare professions)—including the status of the existing literature on gender and race intersectionality. Additionally, we identified strategies, tools, and approaches developed and recommended to address intersectionality in the workplace in the literature interpreted, and summarized findings using numerical and thematic analyses, identified gaps in the literature, and offered recommendations.

## 3. Results

### Descriptive Results

**Figure 2:**
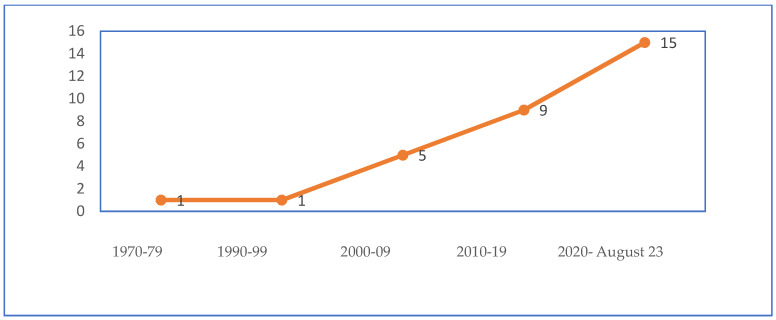
Articles published by year.

**Figure 3:**
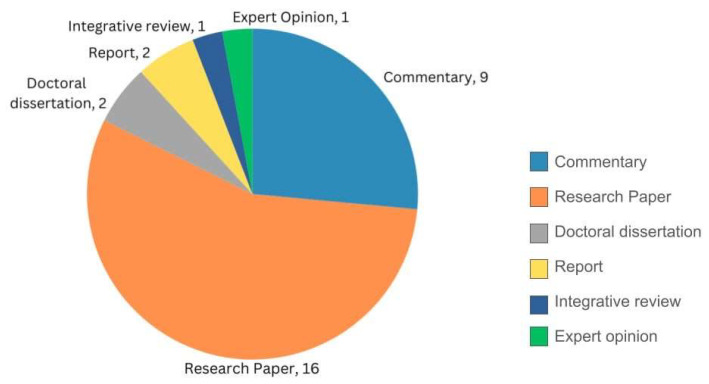
Articles by type of publication.

The search strategy yielded 120 articles for screening, of which 86 articles did not meet the inclusion criteria, thereby providing 33 articles for review. Out of the 33 articles, 2 articles were excluded per the exclusion criteria, and a total of 31 articles were included in the final scoping review [9,19,20,21,22,23,24,25,26,27,28,29,30,31,32,33,34,35,36,37,38,39,40,41,42,43,44,45,46,47,48] (see Table 1). Based on year of publication, the number of articles included in the scoping review were distributed as follows: 1 (3.3%) was published between 1970–1979, and another 1 (3.3%) between 1990–1999; 5 (16.3%) between 2000–2009; 9 (29.0%) between 2010–2019; and 15 (48.3%) were published between 2020–August 2023 (see Figure 2). Among the 31 papers selected for review, 1 (3.3%) was from WHO, 2 (6.6%) were from the United Kingdom, 1 (3.3%) was from New Zealand, and another 1 (3.3%) from North America (USA and Canada), while the remaining 26 (83.5%) were from the U.S. Of the 31 publications included in the scoping review, 4 (13.2%) articles were from the gray literature (i.e., doctoral theses and reports) whereas the remaining 27 (86.8%) articles were peer-reviewed papers (see Figure 3). Of all the papers selected, there was an integrative review, and an expert perspective paper. A total of 2 (6.6%) were reports; another 2 (6.6%) were doctoral theses, while 9 (27.4%) were commentaries, and 16 (52.8%) were research studies. A total of 4 of the 16 research studies analyzed secondary data whereas the remaining 12 collected and analyzed primary data.

**Figure 4:**
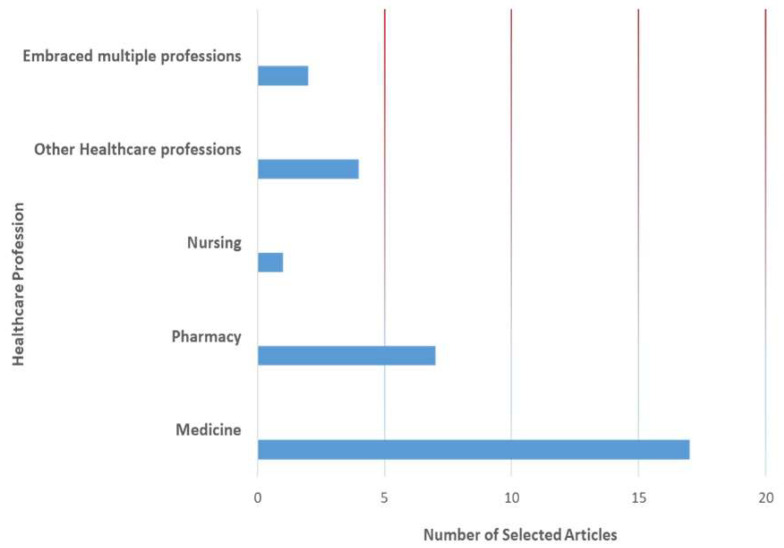
Articles by healthcare profession.

Articles were distributed across the different health professions, including medicine (17), pharmacy (7), nursing (1), other healthcare professions (4), and articles including multiple health professions (2) (see Figure 4). A total of 11 articles addressed academic faculty, 2 articles focused on students, 4 articles included members of professional associations, and the remaining 14 articles focused on healthcare professionals. Key findings in 15 publications demonstrated and/or highlighted (e.g., commentaries) underrepresentation of women and ethnic/racial minority groups in various healthcare professions, including medicine, nursing, and pharmacy. Underrepresentation was shown in membership of professional bodies (one), leadership (five); higher ranks within profession or in academia (three), receipt of awards (one), and in academia of various health professions (eight). Findings from 16 publications showed bias and/or inequities by gender and race (see Table 1). Eleven articles used an intersectional lens [19,20,21,22,23,24,25,26,27,28,29]. There were inequities/biases associated with career progression, rate of promotion, admission into health professions, and remuneration.

Two articles provided historical context of racial and gender inequities in pharmacy [25] and the medical profession [30], respectively. Five publications highlighted challenges associated with professional development and career advancement for WoC [19,20,21,31,32]. One article proposed an interventional framework for enhancing professional wellbeing of women physicians, including those of color [20]. Two articles highlighted the challenges to career progression [21,32]: one looked specifically at Black women [21]; while one article reported more dissatisfaction by race and gender consequent to several factors such as inequities and lack of support [33]. One article demonstrated how intersectionality of identities influences professional identity formation (PIF) for student pharmacists from underrepresented groups (URGs) [27]. One study highlighted the achievements of African American women in pharmacy [23].

## 4. Discussion

This scoping review provided insights into the framing of diversity, representation, and opportunities for advancement for WoC in the healthcare literature. Of the 31 articles in this review, 6 were written in the last 3 years (2020–2022), an indication that inequities at the intersection of race and gender among healthcare professions are yet to be fully explored. In analyzing the articles, several themes emerged that we have broadly categorized as follows:

Category I: Barriers and Challenges

- Underrepresentation;
- Intersectional lens/approach;
- Equity;
- Professional support and networks;
- Leadership and mentoring;
- Sexual harassment and misconduct.

Category II: Opportunities and examples

- Retention and attrition;
- Improving diversity.

### 4.1. Category I: Barriers and Challenges

#### 4.1.1. Underrepresentation

A total of 12 of the 31 articles [21,22,31,34,35,36,37,38,42,43,47,48] addressed historical and current underrepresentation of women and/or minorities. Two articles focused on professional societies and noted underrepresentation of minority women [34,38]; whereas seven articles noted underrepresentation of minority women across different academic disciplines, including dermatology [36]; academic surgery [31]; academic medicine [21,22,42]; family medicine [43]; and pharmacy [48]. Articles underscored persistent underrepresentation of minority women in leadership positions [21,35,37,42,48] across all the different healthcare disciplines. They also note that WoC and some ethnic minorities are likely to be over-represented in lower-paying occupations such as orderlies or nurses, and that ethnic minorities and especially WoC are less likely to be adequately represented in higher paying occupations, a finding that is consistent with the IOM and other reports presented in this paper.

#### 4.1.2. Intersectional Lens/Approach

A total of 19 of the 31 articles included did not apply an intersectional lens but looked at gender and race as separate variables. While inferences regarding women from underrepresented racial minority (URM) groups can be drawn from key findings and/or highlights of these articles, they do not adequately reflect the inequities that affect this population in the healthcare professions. Using an intersectional framework can inform analysis of inequity in outcomes, e.g., workforce retention [30]. Of the 11 articles that applied an intersectional lens, 4 of them were commentaries [19,22,23,25]; and 3 were qualitative interviews [24,26,27]. These, in addition to the expert opinion offered by Ramas and colleagues [20], speak to the exploratory nature of using an intersectional approach to research in this area. The integrative review by Aspinall and colleagues acknowledges the dearth of studies using an intersectional approach, noting that their review is the first to use this approach to investigate issues within the nursing profession, with specific focus on leadership [29]. They also reported that the studies reviewed were mainly qualitative, and generally subject to limitations of design, sampling, and data analysis [29].

#### 4.1.3. Equity

Four of the articles included in the current review investigated inequities in remuneration across race and/or gender. Fruge and colleagues conducted a cross-sectional study of members of the American Society of Pediatric Hematology/Oncology (ASPHO) and found that respondents from underrepresented minority groups reported less access to resources and perceived inequity in salary [33]. Aspinall and colleagues found that poor progression associated with low salary increases for URM women were as a consequence of gender and ethnic discrimination as well as institutionalized racism [29]. In a commentary on experiences of women and underrepresented minority groups in dermatology, the author notes that underrepresented minority women in academia in this discipline had higher clinical burden, and lower remuneration [36]. Umeh’s secondary data analysis of the current population survey (CPS) data reveals that underrepresented minority women in healthcare occupations earn less than their white peers, with the exception of Asians [45].

#### 4.1.4. Professional Support and Networks

The studies that looked at support for women from underrepresented minority groups found that support for this group was often inadequate. The study by Fruge and colleagues found that underrepresented minority respondents reported greater dissatisfaction with organizational support offered by their institution [33]. Furthermore, findings from Parker’s qualitative work investigating the challenges of Black graduate health professional women students point to the critical role of a diverse and supported faculty in enhancing diversity in the student body, as their mentoring of Black students is essential but also an additional burden to their workload [24]. A prospective cohort study by Warner and colleagues found gender and racial disparities in professional network reach, which is a predictor of promotion and retention of medical school faculty [40].

In recognition of the inadequacy of support for women physicians, Ramas and colleagues propose three models to promote well-being and, consequently, the professional advancement of women physicians [20]. These models looked at redefining productivity to capture and acknowledge “invisible” work such as mentoring; redesigning the workplace to facilitate equitable career advancement; and enhancing diversity in leadership. The authors also highlight the need for institutions to further support underrepresented minority women physicians who face other unique challenges [20].

#### 4.1.5. Leadership and Mentoring

The articles reviewed highlight the underrepresentation of underrepresented minority women in leadership in the healthcare professions and the attendant barriers. These include leadership in academic medicine [19,21,35,39]; radiology [37]; family physicians [39]; and nursing [29]. Factors contributing to the racial/ethnic and gender inequity in career progression to leadership positions include implicit bias, systemic racism, and socio-cultural factors. In a commentary by Samra and Hankivsky, they discuss how patriarchal and colonial histories and values have shaped medical education, and the consequent barriers these have created for entry and progression of women and ethnic minorities [30]. They highlight how biases, stereotypes, and implicit assumptions of capabilities have remained constraints to professional progression of ethnic minorities and women [30]. All the articles that touch on leadership agree that these inequities need to be addressed.

In their commentary, Bakken and colleagues highlight the less-than-optimal mentoring experiences of women and URMs with emphasis on gender and/or racial discordance of the mentoring pair and how this impedes career progression [32]. An emergent theme from Parker’s qualitative inquiry captured participants’ experiences with mentoring. They reported that some mentors had to be sought from outside one’s program as there were not enough mentors of the same racial background [24].

#### 4.1.6. Sexual Harassment and Misconduct

Although not mentioned by the other articles, 4 (12.9%) of the 31 articles referred to sexual harassment (and related misconduct) as one of the issues affecting women within some healthcare professions: academic medicine [21,42], pharmacy [25]; with one article addressing healthcare professions in general [9]. For one article, there was access to only the abstract, and so not much can be said about the scope of reporting this issue [25]; however, one study acknowledged sexual harassment as a limitation for women in academic medicine but did not explore it further [21]. One article [42] noted that women were more likely to report sexual harassment and gender discrimination, but only a few cases were reported; and little or no attempt was made by administration to address the situation.

The report by WHO provided a definition for “sexual harassment”, along with further implications for healthcare professionals [9]. For instance, the report stated that while all healthcare workers may face sexual harassment at work, women were more likely to be victimized. Consider that in the United States, 30% of female medical academics reported sexual harassment compared to 4% of men; and of those 30% women who reported harassment, 47% stated that these experiences negatively impacted their career development [9]. The report further states that underreporting of sexual harassment in healthcare professional settings creates a false impression, thereby undermining women professionals and impeding their progress. While this area was not explored in a majority of the articles included in this study, it is an area that warrants further research.

### 4.2. Category II: Opportunities and Examples

#### 4.2.1. Retention and Attrition

With reference to women and minorities, 20 of the 31 articles addressed challenges and interventions related to workforce retentions across various professional fields including: academic medicine [22,31,40,42]; medicine [20,30]; medical clinical research [32]; pharmacy [26,46]; pharmacy education [28]; academic plastic surgery [35]; dermatology [36]; radiology [37]; pediatric hematology/oncology [33]; military family medicine [39]; graduate school health professions [24]; healthcare education and practice [45]; nursing [29]; and healthcare professions [9]. In particular, [9,29,33,36,46] highlighted the influence of female family roles on attrition rates. For instance, unfair choices between career progression and family may cause women to often relinquish their careers [29]. Constraints in balancing paid work with family responsibilities may lead women to either opt out of the workforce [33] or take up part-time work [9]. Additional pressures such as traditional stereotypes of a homemaker further exacerbate this problem as stated in one article, where female pharmacists would take career breaks due to domestic responsibilities [46]. A closely related reason for attrition was noted to be burn-out [36]; for instance, women were more likely to leave academic medicine in 5 years—regardless of academic productivity [36]. Notably, despite the fact that this is an older article from 1999, expectations of domestic responsibilities has not changed by much as discussed in a call to action by Bissell BD, et al. asking organizational leaders to provide sustainable oversight to address gender inequity and sexual harassment [49]. This situation is not unique to the U.S. either, as a Finnish study led by Kuitto K, et al. concluded. Findings from this study helped partly inform the long career breaks that women were more likely to have despite relatively equal labor market participation [50]. Yet another systematic review of the literature (Hawthorne and Anderson) on the global pharmacy workforce noted that even though there was an increase in female participation in the pharmacy workforce, time taken off by females to raise families was greater and likely to result in attrition [51]. The issue of leadership development and further exploration of intersectionality in the pharmacy profession, however, remains a gap in the scientific literature.

Cropsey et al., surveyed 166 participants who had left their academic institution to better understand reasons for leaving. Common reasons for leaving reported by women included, chairman/departmental leadership issues (30.8%), career/professional advancement (29.8%), low salary (25%), and personal reasons (25%). For non-whites, the most common reasons included career/professional advancement (32.4%), low salary (29.4%), and personal reasons (29.4%). Women were significantly less likely to evaluate their opportunity for advancement and rate of promotion as good to excellent compared with their male counterparts [42]. Lewis-Stevenson et al., found in their study that women were one-fourth as likely as men to be full professors and half as likely to be associate professors. Only 9% of faculty members were from underrepresented minorities. The study further reported low odds (0.4) for minority faculty to become senior faculty [43].

While solutions to improve diversity and representation are more likely to focus on recruitment, attention should be paid to the retention of minority professionals, particularly WoC, along with addressing career advancement, leadership attitudes and support, wage/pay gaps, and support systems in place to successfully retain and develop talented individuals. Lack of retention of females is one of the potential causes for the gender gap in promotion [37]. By continuing to propagate inequities, institutions, in turn, only hinder their own abilities to recruit, retain, and keep engaged, talented faculty [35]—especially because most of the main reasons cited for leaving institutions are reportedly avoidable and amenable to intervention [42]. Ramas et al. claimed that certain factors, when in place, would likely improve retention for women (and thrive in) the same work environment [20]. For instance, career development programs may improve female and racial minority retention [39].

Successful mentorship programs can develop faculty who are academically productive, promoted earlier, and more likely to stay at their institution [31]. Having a network of productive colleagues (e.g., level of co-authorship) has been reported to be among the strongest predictors of retention [40]. Another article suggested that having a manager of the same ethnic origin would have a positive effect on decisions for career progression [26], whereas Wong et al. suggest the development of indicators to track retention strategies within academia [22]. It would be imperative, however, to study attrition and declining of a position by WoC to better understand the reasons for attrition and, conversely, factors that may promote or enhance recruitment and retention over longer periods of time.

#### 4.2.2. Improving Diversity

A total of 17 of the 31 articles highlighted the need to increase diversity in healthcare professions, including academic medicine [22,31]; medicine [20]; healthcare education and practice [45]; medical physics [34]; oncology and hematology [33,38]; academic plastic surgery [35]; graduate school health professions [24]; radiology [37]; dermatology [36]; pharmacy [46]; pharmacy education [27,28]; academic pharmacy [48]; and healthcare professions [9,47]. Several articles stated that concordance of race and gender was critical to provide care for diverse populations [35]. For instance, physician–patient racial/ethnic concordance has been associated with better communication, increased patient participation in decision-making, and improved adherence to medical advice [36]. Increasing diversity can foster innovation, greater financial efficiency, and improve patient outcomes [37]. In particular, attention to diversity within educational environments was highlighted [24,28,45]—emphasizing how the presence of minority faculty can lead to more inclusive and effective learning experiences for both faculty and students. This is particularly relevant in the light of the IOM and Sullivan reports [1,2] presented earlier in this paper regarding the diversity of not just healthcare professionals but also faculty and students. While the benefits of a diverse workforce have been well explored, the recruitment and retention of talented professionals and WoC remains challenging.

Strategies for recruitment and retention of WoC need institutional commitment, leadership support, and deliberate planning. Strategies may include developing indicators that track diversification of faculty [22]; reporting gender and racial disparities in various areas of academia [38]; developing a diverse leadership team by intentionally promoting more WoC into first-level management [20]; and offering wellness-oriented models to promote professional fulfillment and well-being [20].

**Case Study: Pharmacy**

Seven studies [25,26,27,28,46,47,48] addressed pharmacy health professionals, of which Queneau [47] examined occupational patterns of occupational segregation by race and ethnicity in healthcare for 16 healthcare professions (including pharmacy). The pharmacy profession has been experiencing demographic shifts in the past few decades, particularly in the US and UK. Recent data have shown an increase in WoC in the pharmacy workforce in the US [52], UK [53,54], and Canada [55]. Platts et al. described in 1999 the feminization of the pharmacy profession and described the profession as being in transition. They further implied that acceptance of flexible working patterns, childcare availability, and increasing numbers of ethnic minorities in pharmacy, necessitated that the profession be proactive in its recruitment and flexible with its dynamic nature [46]. The pharmacy profession has become one of the most attractive professions to women due to its flexible working and part-time hours, and general working conditions. Despite the growing numbers of women pharmacists of color, there is little empirical research on the experiences, professional development, and advancement of WoC. More work must be achieved to demonstrate the profession’s commitment to diversity, beginning with student recruitment at colleges of pharmacy [26]. Hahn et al. [28] explored career engagement, interest, and retention of minority students at multiple schools and colleges of pharmacy and found that participants were most confident in their ability to obtain a job in community or hospital pharmacy but least confident about academic teaching or the pharmaceutical industry. While the study sample was small and not generalizable, the dearth of WoC in academic teaching needs to be addressed. Similarly, Rockich-Winston et al. [27] found that intersectionality of identities created advantages in belonging to some social categories and disadvantages in belonging to others for student pharmacists who are developing their professional identities. Chisholm-Burns et al. [48] noted the lack of women in leadership positions, citing that only 18% of all hospital CEOs were women, and in the healthcare sector, women leaders accounted for a mere 25%. Though it has been noted that inclusion of women in business leadership significantly increases firm value, financial performance, economic growth, innovation, social responsibility, and capital, such inclusion continues to be low in the healthcare professions. The article addressed challenges and barriers to professional development of women and presented strategies identified by the American Society of Health-Systems Pharmacists (ASHP) Women in Pharmacy Leadership Steering Committee that includes above all, soul-searching and reflection by the pharmacy community to make concerted efforts to achieve equality in compensation and representation of women in pharmacy. A yet-to-be-addressed area is the prospect of unionization of pharmacists, particularly women, since unions tend to be predominantly male dominated. However, the lower numbers of women in leadership positions make it challenging for women to unionize even though they may benefit from collective bargaining. Possibly, such unionization may be likely to occur within homogenous workplaces and unions, and, when available, ought to offer training and mentoring programs for WoC [47]. Lastly, Abdul-Muktabbir et al. used the term “intersectional invisibility” to describe the marginalization experienced by Black, Indigenous, and persons of color (BIPOC) women and the harms perpetuated by single-axis movements that fail to take into account the experiences of discrimination of BIPOC women and the difference from minoritized men [25].

### 4.3. Research Gaps and Areas for Future Work

Through the scoping review exercise, we identified several areas that need to be better understood, developed, and explored as discussed below:

#### 4.3.1. Research Mostly Exploratory

The articles reviewed were mostly exploratory, and along with the commentaries, make the case for further research in this area. While these provide pertinent data that clearly demonstrate the persistence of inequities that disparately affect women from underrepresented minority racial/ethnic groups, they do not quantify these disparities and do not provide the depth of insight needed to fully understand the implicated systemic factors and adequately address those structurally. Of note is the dearth of research in the literature pertaining specifically to the pharmacy profession, indicating a gap in research in this disciplinary area. Given the increase in the prevalence of women in the pharmacy profession, it is critical that women from minoritized racial/ethnic groups are not at a disadvantage professionally and otherwise, hence, the need for research in this regard.

#### 4.3.2. Limited Use of Intersectionality

The current review of the literature also demonstrates the limited use of intersectionality as a framework for understanding and addressing barriers to well-being and professional advancement of women from underrepresented racial/ethnic groups in the healthcare professions. Intersecting identities result in unique lived experiences, opportunities, barriers, and facilitators that may differ markedly from those of persons who share one of those identities. For example, a woman of color may experience gender-based disadvantages and challenges, such as salary inequity, along with her white counterparts when compared to men in similar positions. However, she will have a unique experience based on the intersection of her gender and racial/ethnic identities.

#### 4.3.3. Non-Representative Sampling

There were 18 research articles reporting primary and/or secondary data analysis. Six of these looked at women from underrepresented minority groups as a distinct group(s) [21,24,26,27,28,29]. Ten articles conducted cross-sectional surveys [21,28,33,34,35,39,42,43,46,48]; with two also including interviews [21,46]; and one with free text survey items in a mixed-methods approach [48]. For all the research studies, sampling was convenient and/or purposeful, and, therefore, limited in generalizability to their respective study populations.

#### 4.3.4. Aggregation across Groups

Three studies aggregated data across groups [35,43,45]. In other cases, minoritized racial/ethnic groups were captured as one category, for example all non-white referred to as underrepresented minorities [33]. While there may be commonalities in some of the barriers experienced across groups, there are differences in lived experiences, historical contexts, and how each group is perceived in the broader society. Further research should, therefore, look at each group as a distinct entity.

#### 4.3.5. Variability in Terminology and Classifications

This review included articles on inequities in healthcare professions by gender and race/ethnicity with a focus on WoC (i.e., women from minoritized racial/ethnic groups). However, in reviewing the articles, the researchers found that different terms are used, some of which refer to racial/ethnic populations and others to underrepresented groups that may be inclusive of other minoritized identities in addition to race/ethnicity. There were arbitrary assignments of gender and race categories in some studies, without any explicit definition. For example, one study pointed out that they only had binary assignments for gender that failed to capture the full gender spectrum of self-identities of minority professionals [38]. The variability in the definitions and classifications of both gender and race/ethnicity is problematic in summarizing the evidence base regarding inequities that affect WoC in the healthcare professions.

#### 4.3.6. Generalizability

Articles that reported original research were studies that employed cross-sectional survey methods and/or qualitative methods of inquiry, with sampling performed for convenience, thereby limited in their generalizability to the respective study population. Response rates, where reported, were low. Furthermore, findings in one healthcare profession may not be applicable to other professions or across geographic locations or organizations. However, the findings provide insight that should inform further investigation.

Assumptions and biases pertaining to women and minorities are not only deeply morally troubling but also impediments to the growth of the health care professions [48]. Of the approximately 333 million people living in the U.S, 50.4% are women, 41.1% are non-whites (including Hispanic and Latino), and 17.3% are over 65 years of age [5]. The increase in age expectancy and increasing elderly population along with changing demographics necessitate that the healthcare workforce is adequately prepared to deal with the challenges of an aging and diverse multicultural population.

##### Limitations

Our scoping review used strict inclusion/exclusion criteria that may have excluded some articles addressing minorities and women but not both in the context of the healthcare professions. We also excluded articles not written in English that may have limited the number of papers available for the study. We did not have access to the full text for three of the articles [25,41,43] included in the review, necessitating the review of the respective abstracts. The determination to include the studies was based on how detailed the abstracts were in providing relevant information.

## 5. Conclusions

This scoping review reveals that WoC have unique and distinct challenges and barriers in their professional careers resulting from the intersectionality of not only race and gender, but also their lived experiences and opportunities. The healthcare professions are in a period of transition. Demographic shifts in the population as well as in the workplace necessitate a deeper understanding of the unique challenges and barriers faced by WoC in achieving their professional goals. From admissions in academic institutions and training, to recruitment, retention, development opportunities, supportive leadership and networks, and institutionalized discrimination, WoC have distinct challenges that must be addressed to improve diversity and representation, particularly in leadership and management positions. In the last few years, much attention and effort has been directed to recruiting WoC in higher positions in efforts to increase diversity and mitigate institutionalized discrimination. However, as the results from this scoping review indicate, it is not merely enough to recruit, but rather efforts should be directed at retaining and developing WoC to achieve higher-level positions. Such efforts should be directed at addressing the distinct challenges that WoC face, including sexual harassment, stereotyping, discrimination, lack of institutional investment and leadership support, wage/pay gaps, lack of professional networks, and mentoring.

Strategies presented from the literature to mitigate institutionalized discrimination included prioritizing mentoring and early career education/sponsorship; creating support networks and allocating resources to developing avenues of support; professional and formal leadership development programs; expanding promotion criteria to include diversity work and community service; closing the gender pay-gap; advocacy and support from non-minority authorities/institutions; creating minority-based professional societies; improving representation on journal editorial boards; conducting regular assessments/surveys regarding perceived mistreatment; raising awareness of implicit/explicit bias; and identifying elements in the physical environment (like portraits) that might contribute to inequity. Whether such strategies when implemented in a concerted manner with intentionality serve to improve diversity and representation in the healthcare professions remains to be seen.

Therefore, future research in this field would be served by using an intersectional lens or framework to develop and monitor any strategies to address professional development of WoC in healthcare along with critical analyses of their outcomes.

## Figures and Tables

**Table 1:** Overview of the data extracted from the 31 articles.

*Authors, Year of Publication*	*Article Type, Study Design (If Applicable)*	*Study Population*	*Area of Focus*	*Key Relevant Findings*
*^a^ Rankin et al., 2023 [34]*	Research; Cross-sectional, Secondary data analysis	The 2020 American Association of Physicists in Medicine (AAPM) membership	Gender and racial diversity/representation (professional membership)	– Moderate increase in gender and racial diversity in professional membership (2002–2020) – Underrepresentation of Women, Hispanic/Latinx/Spanish individuals, and individuals reporting a race other than white or Asian
*^a.^ Chawla et al., 2021 [35]*	Research; cross-sectional, Secondary data analysis	Academic faculty with (1) an MD or equivalent, (2) academic ranking, (3) plastic surgery training, and (4) accredited plastic surgeons	Gender and racial inequity (leadership, scholarly productivity)	– Underrepresentation of women of color in faculty leadership in North America – Less representation of women and underrepresented minorities in leadership in the US compared to Canada
*^a,b^ Okoye, 2020 [36]*	Commentary	Women and underrepresented minorities in medicine (UIM) in dermatology	Unique experiences and/or challenges	– Underrepresentation of UIM women in academic dermatology – Compared with their majority colleagues, UIM women in academia have higher clinical burden, and lower remuneration
*^b,c^ Verduzco-Gutierrez et al., 2022 [19]*	Commentary	Women of color in academic medicine	Unique experiences and/or challenges women of color	– Institutional gender bias as a barrier to progression of women of color to leadership in academic medicine – Potential strategies and recommendations
*^c^ Ramas et al., 2021 [20]*	Expert opinion/perspectives	Women physicians	Gender inequity (rate of promotion and career advancement)/ unique experience and/or challenges (professional fulfillment and well-being)	– Three wellness-oriented models are presented to promote the professional fulfillment and well-being of women physicians – Highlights intersectionality (race and gender) and emphasizes the need for more tailored support for under-represented minority (URM) women physicians (by race/ethnicity and gender identity)
*^a^ Manik and Sadigh, 2021 [37]*	Commentary	Women and underrepresented minorities in medicine (radiology)	Gender and racial diversity/representation (education, leadership, research, and workforce)	– Underrepresentation of women of color in leadership in radiology – Decreasing proportion of women and minorities represented in radiology with increasing rank or job title elevation
*^a^ Patel et al., 2021 [38]*	Research; retrospective, observational study	Awards recipients in oncology and hematology	Gender and racial representation (within award recipients)	– Underrepresentation of women and persons from minority groups among award recipients from the seven major international hematology and oncology societies in the world
*^b^ Massaquoi et al., 2021 [39]*	Research; cross-sectional survey	Registered attendees of the 2016 Uniformed Services Academy of Family Physicians	Gender and racial inequity (academic medicine and healthcare leadership) Gender and racial representation (attaining early career leadership positions)	– Inequity in leadership positions and opportunities for advancement between Caucasians and Non-Caucasians or males compared with females
*^a^ Newman et al., 2019 [31]*	Report; assessment and programmatic initiatives	Women and minorities in academic surgery	Gender and racial diversity/representation (professional fulfillment and career success).	– Persisting underrepresentation of women and significant absence of under-represented minority faculty in academic surgery
*^a,c^ Hill et al., 2016 [21]*	Research: mixed Methods; interviews, survey (*description of study design; no findings reported*)	Women of color junior faculty in academic medical institutions	Gender and racial diversity/representation Unique experiences and/or challenges (institutional, individual, and sociocultural factors that influence the entry, progression, and advancement of women of color in academic medicine)	– Underrepresentation of women of color among senior biomedical scientists and academic medical faculty as rationale – Study aims to identify the factors implicated in career progression and leadership attainment
*^b^ Warner et al., 2015 [40]*	Research; prospective cohort study	Medical School faculty with rank of assistant or associate professor	Gender and racial inequity (predictors of intra-organizational connections measured by network reach; and their associations with promotion and attrition)	– Minority (African American, Hispanic, and Native American) and women faculty members had lower network reach; higher network reach was associated with likelihood of promotion and less likelihood of leaving the institution
*^b^ Fruge et al., 2011 [33]*	Research; cross-sectional survey	American Society of Pediatric Hematology/Oncology (ASPHO) members	Gender and racial inequity Unique experiences and/or challenges (comparative career pathway experience of women and minority ASPHO members)	– More dissatisfaction reported among minority and women respondents – Less access to resources and perceived inequity in salary reported among minority respondents – More dissatisfied with work–life balance and organizational support offered reported among minority respondents – Women and minority respondents reported negotiating less successfully
*^b^ Pololi and Jones, 2012 [41]*	Research (qual); interviews	Medical faculty representing various disciplines at four different career stages (early career, leaders, plateaued, and left academic medicine)	Gender inequity, unique experiences and/or challenges (marginalization)	– Women had a sense of “not belonging” in the organization, self-perception of being an “outsider”, feeling isolated and invisible – Barriers to advancement, including bias and gender role expectations – Perception of double disadvantage among faculty from underrepresented minority groups and PhDs
*^a,b^ Cropsey et al., 2008 [42]*	Research; survey	Medical school faculty who left the school of medicine	Unique experiences and/or challenges (women and minority faculty attrition)	– Underrepresentation of women and non-white faculty in higher professional ranks – Women and non-white faculty are more likely to be at lower ranks (instructor or assistant professor) – Lower rating of career progression and rate of promotion – Women significantly less likely to evaluate their opportunity for advancement and rate of promotion as good to excellent compared with their male counterparts
*^b^ Bakken et al., 2006 [32]*	Commentary	Physician scientists	Unique experiences and/or challenges (career progression, mentoring, performance)—women and underrepresented minorities	Highlights the unique challenges to career progression for women and underrepresented minorities: – Less than optimal mentoring experience with gender and/or racial discordance – Impact of gender and racial stereotypes on performance
*^a,c^ Wong et al., 2001 [22]*	Commentary	Underrepresented minority (URM) physician faculty	Gender and racial representation. (initiative to increase URM faculty recruitment)	Highlights persisting underrepresentation of women of color among medical school faculty and describes efforts to increase representation
*^a,b^ Lewis-Stevenson et al., 2001 [43]*	Research; survey	Women and minority physician faculty in departments of family medicine.	Gender and racial inequity (role and academic positions of women and minorities)	– Gender inequity in likelihood of becoming associate or full professors – Underrepresentation of persons from minoritized racial groups – Racial inequity in likelihood of becoming senior faculty
*^b^ Weaver and Garrett, 1978 [44]*	Commentary	Women and URM health professionals	Gender and racial inequity, unique experiences and/or challenges (women and URMs as candidates for professional schools, health care workers/providers, and service users).	Highlights: – Gender and racial inequity in health professions admissions – Discrimination against women and minorities in the health care professions – Distinction of sexism vs. racism in the context of the healthcare industry
*^b,c^ Clark, 2022 [23]*	Commentary	African American (AA) women in professional pharmacy associations	Unique experiences and/or challenges (roles in professional pharmacy associations between 1900–1970)	Highlights – Black women’s achievements in professional pharmacy associations: addressing injustices and advocating for civil rights contributory to paving the way for inclusion and equity
*^c^ Parker, 2020 [24]*	Doctoral thesis research (qual); interviews	African American and African graduate health professional women students	Unique experiences and/or challenges (Black women who had gained entry to or completed graduate education in the health professions)	Emergent themes reflected unique challenges of Black women, including: – Some mentors are inherent/others must be sought out – Experiences and forward-thinking reinforcement matter – Sense of security matters – Student diversity starts with a diverse and supported faculty – Issues both in and outside of school must be addressed – Inclusion must be genuine and meaningful – There is power in being heard
*^b^ Umeh, 2012 [45]*	Doctoral thesis: research; secondary data analysis	Women working in health professions and aged 18–65; 2008–2010 CPS data	Gender and racial inequity (income earned—non-white women, women with children ≤ 6 years old, immigrant women)	Gender and racial inequity in pay—minority women who work in health care occupations earn less annually than their white counterparts, with the exception of Asians
*^b,c^ Abdul-Mutakabbir et al., 2022 [25]*	Commentary	Black, Indigenous, and persons of color (BIPOC) women in pharmacy	Gender and racial inequity (historical context) Unique experiences and/or challenges (BIPOC women in pharmacy)	Highlights historical context of racism and gender inequity
*Platts and Tann, 1999 [46]*	Research (mixed methods); interviews, survey	Ethnic minority pharmacists and non-ethnic minority pharmacists (registered pharmacists)	A comparative analysis; unique experiences and/or challenges (female and ethnic minority pharmacists—roles, career aims, and outcomes)	Differences in career trajectory and career expectations between female CPh (control pharmacist—non-ethnic), and female EPh (ethnic and minority pharmacists) – With increasing age, CPh tended to move away from full-time employment towards part-time employment, while EPh either left the profession or became owners – EPh had high levels of ambition for promotion, but their perceptions of likelihood of success were low
*^c^ Howells et al., 2018 [26]*	Research (qual); interviews	Women from Black, Asian, and minority ethnic groups (BAME) and white women pharmacists	A comparative analysis; unique experiences and/or challenges (choices and work patterns)	– Career trajectories and opportunities similar for women part-time workers irrespective of ethnic origin – Normative factors (such as cultural ideals and parental expectations about medical and pharmacy careers) likely critical influences on BAME women’s pharmacy sector preferences
*^c^ Rockich-Winston et al., 2023 [27]*	Research (qual); interviews	Student pharmacists from underrepresented groups (URGs)	* Unique experiences and/or challenges (professional identity formation (PIF))	– Intersectionality of identities results in perceptions of advantages belonging to certain social categories, while simultaneously being disadvantaged belonging to other social categories – Intersectionality influences professional identity formation (PIF) for student pharmacists from underrepresented groups (URGs)
*^a^ Queneau, 2006 [47]*	Research; secondary data analysis	The healthcare workforce is represented in 16 occupations, representing ~ 90 percent of total employment in the healthcare workplace.	Gender and racial representation (patterns of occupational segregation by gender and race–ethnicity in healthcare)	– Increased representation of women in higher-paying occupations such as physicians, dentists, and pharmacists; but persisting underrepresentation in such occupations over the period 1983–2002 – Over-representation of women and Black people in low-paying occupations such as nursing aides, orderlies, and attendants. – Underrepresentation of Black and Hispanic people in better-rewarded occupations
*^a^ Chisholm-Burns et al., 2012 [48]*	Research (mixed methods); survey with open- and closed-ended questions.	Female, full-time faculty members of a public non-historically Black colleges and universities (HBCU) college or school of pharmacy	Gender and racial representation (trends in the numbers of women and underrepresented minority (URM) pharmacy faculty) Unique experiences and/or challenges (factors influencing academic career pursuit and retention)	– Persisting underrepresentation of URM women pharmacy faculty members at each rank and administrative (i.e., Dean) position
*^a,b^ World Health Organization, 2019 [9]*	Report	The global healthcare workforce	Gender representation (trends and dynamics in the health workforce)	– Acknowledges gender inequality in health and social care workforce globally – Highlights gaps in data and research
*^c^ Hahn et al., 2021 [28]*	Research; cross-sectional survey	Doctor of Pharmacy (PharmD) students identifying as underrepresented racial minorities (URMs)	Unique experiences and/or challenges (pharmacy career engagement, interest, and confidence URM PharmD students)	– Female Doctor of Pharmacy (PharmD) students identifying as underrepresented racial minorities (URMs) more likely than males to report having frequent exposure to community pharmacy during school – Doctor of Pharmacy (PharmD) students identifying as underrepresented racial minorities (URMs) most confident in their ability to obtain a job in community pharmacy (vs. hospital and residency)
*^b,c^ Aspinall et al., 2023 [29]*	Integrative review	The nursing profession	* Gender and racial inequity (nursing leadership)	– Gender gap in global health leadership, resulting in a male-dominated yet feminized sector – Ethnic and gender discrimination (unconscious bias and institutional racism) result in poor progression with associated low salary increases
*^b^ Samra and Hankivsky, 2021 [30]*	Commentary	The medical profession	Gender inequity (impact of patriarchal cultures and colonial histories and values)	Highlights – How patriarchal and colonial histories and values have shaped medical education; constraining women doctors’ career choices and progression internationally – Implicit and explicit biases based on social stereotyping that shape the identification, cultivation, and selection of individuals chosen for programs and internships – How unconscious bias can contribute to systematic underestimation of the capabilities of qualified women and ethnic minority and internationally trained applicants – The need to recognize and challenge Whiteness norms and patriarchal practices in medicine

^a.^ Article demonstrating underrepresentation by gender and race/ethnicity; ^b.^ article demonstrating bias/inequity by gender and race/ethnicity; ^c.^ article using an intersectional lens.

## Data Availability

The data presented in this study are available in Table 1.
